# Microvessel Density and Status of p53 Protein as Potential Prognostic Factors for Adjuvant Anthracycline Chemotherapy in Retrospective Analysis of Early Breast Cancer Patients Group

**DOI:** 10.1007/s12253-012-9525-9

**Published:** 2012-05-02

**Authors:** Beata Biesaga, Joanna Niemiec, Marek Ziobro

**Affiliations:** 1 Department of Applied Radiobiology, Centre of Oncology, Garncarska 11, Kraków, 31-115 Poland; 2 Department of Medical Oncology, Centre of Oncology, ul. Garncarska 11, Krakow, Poland

**Keywords:** Early breast cancer patients, Anthracyclines, Angiogenesis, p53 status, Potential prognostic factors

## Abstract

A considerable subgroup of patients with early breast cancer does not address benefits of anthracycline based chemotherapy. The aim of this retrospective study was to investigate the effect of microvessel density (MVD) and status of p53 protein on 5-year disease free survival (DFS) in the group of breast cancer patients treated with anthracyclines in adjuvant setting. Correlations between MVD, p53 status and other clinicopathological parameters were also assessed. MVD and p53 status were analyzed immunohistochemically in the group of 172 women with breast cancer in clinical stage T1-2, N1-N2, M0. There were 123 tumors (71.5 %) with lower MVD (≤214.8 microvesells/mm^2^) and 49 (28.5 %) with higher MVD (>214.8 microvesells/mm^2^). The proportion of higher MVD tumors significantly increased in N2 (*P* = 0.000) and in estrogen (*P* = 0.046) or progesterone receptors (*P* = 0.029) negative tumors. p53 positivity was indicated in 50 cancers (29.1 %) and was significantly associated with higher grade (*P* = 0.000), steroid receptors negativity (*P* = 0.000), cytokeratin5/6 positivity (*P* = 0.026), topoisomerase IIα overexpression (*P* = 0.005) and higher proliferation rate (*P* = 0.001). In univariate analysis, higher MVD (*P* = 0.016) and p53 negativity (*P* = 0.023) were significantly related with longer DFS (median follow-up 36 months). In multivariate Cox regression analysis MVD was independently associated with DFS. These data suggest that higher MVD is favourable prognostic factors for early advanced breast cancer patients after adjuvant anthracycline based chemotherapy.

## Introduction

The clinical outcome of node positive breast cancer is heterogeneous despite administration of adjuvant systematic chemotherapy with anthracyclines [1, 2]. Therefore, there is a need for identification of the patients with good prognosis who do not need further adjuvant treatment and those with worse prognosis, for whom alternative, non-anthracycline containing regimens must be applied. The available prognostic parameters (lymph node status, tumor size, grade of malignancy, expression of steroid receptors and human epidermal growth factor receptor type 2 (HER2)) do not define the prognosis of individual patient after anthracycline treatment precisely. Hence extensive research has been carried out to identify novel prognostic factors for a better selection of the optimal treatment. In the previous retrospective study we have shown that all patients (*n* = 52) with G1 + G2 tumors without topoisomerase IIα (TOPOIIα) overexpression survived 5 years without progression after completing anthracyclines treatment [3]. The worst prognosis (DFS: 66.7 %) was found for patients with TOPOIIα overexpressed and G1 + G2 tumors. Thus, there is still a need to search for new prognostic factors to identify patients with high risk of metastases development.

Angiogenesis is essential for the growth of both primary and metastatic tumors. To assess microvessel density, Weidner et al. [4] suggested identification in tumor specimens of areas with the highest number of microvessels. They also reported in the group of 49 patients with primary invasive breast carcinoma, that higher MVD was associated with axillary lymph nodes or distant metastases. Since that time, many studies [5, 6] as well as meta-analysis [7], have shown that low MVD is related with good prognosis in breast cancer patients with negative axillary lymph nodes. However, the role of angiogenesis as a prognostic factor for adjuvant anthracycline based chemotherapy is unclear in women with positive lymph node. In this cohort some reports [8–10] suggest an association between low MVD and good prognosis. By contrast, other studies [11–15] failed to confirm this hypothesis, while in several analyses [16–18] reverse relation has been found.

Experimental evidence indicates that p53 protein, a product of tumor suppressor gene, which is a key regulator of cell response following DNA damage by anthracyclines, additionally influences angiogenesis. p53 contributes to angiogenesis regulation in two ways: it supports the secretion of inhibitory thrombospondin-1 and depresses the secretion of the vascular endothelial growth factor (VEGF) inducer [9]. Although experimental studies have clearly shown that p53 mutated form confers resistance to anthracyclines [19, 20], translational studies have demonstrated non-concordant data. Several reports [21–24] showed that p53 overexpression assessed by immunohistochemistry (IHC) correlated with resistance to anthracyclines, nevertheless other studies suggested lack of such relation [25–27].

There are two main causes of these discrepancies in the results of translational researches in the case of both biomarkers: lack of standardized methods for MVD or p53 status evaluation and differences between these studies considering patient selection (N + or/and N−), number of patients enrolled, use of different polychemotherapy regimens and length of follow-up. Thus, the prognostic role of MVD and status of p53 protein in specific subgroups of breast cancer patients remains controversial. Therefore, in this report we analyzed, according to our best knowledge for the first time in so large and homogenous with respect to tumor clinical stage and adjuvant chemotherapy type, the relation between MVD or p53 status and DFS in the group of patients with T1-T2, N1-N2, M0 breast carcinoma, treated with anthracyclines in adjuvant setting. Furthermore, we investigated the correlations between MVD or p53 status and other assessed previously clinicopathological variables including tumor clinical stage, grade, estrogen (ER) and progesterone (PgR) receptors, HER2, cytokeratin 5/6 (CK5/6) status as well as TOPOIIα expression and proliferation rate.

## Material and Methods

### Patients

A series of 172 patients with operable (T1-2, N1-2, M0) invasive primary breast carcinoma treated with adjuvant anthracyclines based chemotherapy was identified between 2001–2005 at Centre of Oncology, Krakow Branch, Poland. Details regarding the study population, inclusion and exclusion criteria, treatment type, immunohistochemical analysis of steroid hormone receptors, CK5/6 and HER2 status, TOPOIIα expression (by TOPOIIα labelling index (TOPOIIALI)) and proliferation rate (by Ki-67 labelling index (Ki-67LI)) have been presented previously [3]. Table 1 summarizes all details concerning clinical and histopathological characteristics of patients involved in this study.

**Table 1:** Clinical and histological features of breast cancer patients stratifying according to MVD and p53 status

	All	MVD		*P-*value^a^	p53LI		*P-*value^a^
		≤ 214.8 vessel/mm^2^	> 214.8 vessel/mm^2^		<= 10 %	> 10 %	
	N (%)	N (%)	N (%)		N (%)	N (%)	
Number of tumours	172 (100.0)	123 (71.5)	49 (28.5)	NS	122 (70.9)	50 (29.1)	NS
Age:							
<50 years	57 (33.1)	45 (78.9)	12 (21.1)	NS	38 (66.7)	19 (33.3)	NS
≥50 years	115 (66.9)	78 (67.8)	37 (32.2)		84 (73.0)	31 (27.0)	
Tumour size:							
T1	53 (30.8)	38 (71.7)	15 (28.3)	NS	40 (75.5)	13 (24.5)	NS
T2	119 (69.2)	85 (71.4)	34 (28.6)		82 (68.9)	37 (31.1)	
Nodal status:							
N1	133 (77.3)	108 (81.2)	25 (18.8)	0.000	92 (69.2)	41 (30.8)	NS
N2	39 (22.7)	15 (38.5)	24 (61.5)		30 (76.9)	9 (23.1)	
Grade:							
G1 + G2	92 (53.5)	64 (69.6)	28 (30.4)	NS	80 (87.0)	12 (13.0)	0.000
G3	80 (46.5)	59 (73.8)	21 (26.2)		42 (52.5)	38 (47.5)	
Type of surgery*:*							
Mastectomy	153 (89.0)	112 (73.2)	41 (26.8)	NS	110 (71.9)	43 (28.1)	NS
BCS	19 (11.0)	11 (57.9)	8 (42.1)		12 (63.2)	7 (36.8)	
Type of adjuvant chemotherapy:							
AC:	103 (59.9)	62 (60.2)	41 (39.8)		84 (81.5)	19 (18.5)	
4 series	51 (49.5)	30 (58.8)	21 (41.2)	NS	44 (86.3)	7 (13.7)	NS
6 series	52 (50.5)	32 (61.5)	20 (38.5)		40 (76.9)	12 (23.1)	
CAF:	69 (40.1)	61 (88.4)	8 (11.6)		38 (55.1)	31 (44.9)	
4 series	5 (7.2)	3 (60.0)	2 (40.0)	0.043	3 (60.0)	2 (40.0)	NS
6 series	64 (92.8)	58 (90.6)	6 (9.4)		35 (54.7)	29 (45.3)	
Estrogen status:							
*Positive*	127 (73.8)	95 (74.8)	32 (25.2)	0.046	104 (81.9)	23 (18.1)	0.000
*Negative*	45 (26.2)	28 (62.2)	17 (37.8)		18 (40.0)	27 (60.0)	
Progesterone status:							
*Positive*	124 (72.1)	92 (74.2)	32 (25.8)	0.029	101 (81.5)	23 (18.5)	0.000
*Negative*	48 (27.9)	31 (64.6)	17 (35.4)		21 (43.8)	27 (56.3)	
Cytokeratin 5/6 expression:							
*Positive*	35 (20.3)	18 (51.4)	17 (48.6)	0.017	18 (51.4)	17 (48.6)	0.026
*Negative*	137 (79.7)	105 (76.6)	32 (23.4)		104 (75.9)	33 (24.1)	
HER2 status:							
*Overexpressing*	68 (39.5)	51 (75.0)	17 (25.0)	NS	47 (69.1)	21 (30.9)	NS
*Not overexpressing*	104 (60.6)	72 (69.2)	32 (30.8)		75 (72.1)	29 (27.9)	
TOPOIIALI^b^:							
≤11.9 %	84 (48.8)	63 (75.0)	21 (25.0)	NS	69 (82.1)	15 (17.9)	0.005
>11.9 %	88 (51.2)	60 (68.2)	28 (31.8)		53 (60.2)	35 (39.8)	
Ki-67LI^b^:							
*≤19.7 %*	98 (57.0)	67 (68.4)	31 (31.6)	NS	79 (80.6)	19 (19.4)	0.001
*>19.7 %*	74 (43.0)	56 (75.7)	18 (24.3)		43 (58.1)	31 (41.9)	
MVD^c^:							
*≤214.8 vessel/mm* ^*2*^	123 (71.5)				86 (69.9)	37 (30.1)	NS
*>214.8 vessel/mm* ^*2*^	49 (28.5)				36 (73.5)	13 (26.5)	
p53LI:							
*≤10.0 %*	122 (70.9)	86 (70.5)	36 (29.5)	NS			
*>10.0 %*	50 (29.1)	37 (74.0)	13 (26.0)				
Breast cancer immunofenotype^d^:							
*Luminal A*	80 (46.4)	58 (72.5)	22 (27.5)	NS	63 (78.8)	17 (21.2)	0.001
*Luminal B*	44 (25.6)	34 (77.3)	10 (22.7)		36 (81.8)	8 (18.2)	
*HER2+*	24 (14.0)	15 (62.5)	9 (37.5)		11 (45.8)	13 (54.2)	
*Basal-like*	24 (14.0)	16 (66.7)	8 (33.3)		12 (50.0)	12 (50.0)	

*MVD* microvessel density, *p53LI* p53 labelling index, *BCS* breast conserving surgery, *AC* doxorubicin, cyclophosphamide, *CAF* cyclophosphamide, doxorubicin, 5-fluorouracil, *TOPOIIALI* topoisomerase IIα labelling index, *Ki-67LI* Ki-67 labelling index, *n.s.* non significant

^a^Pearson *χ* ^2^ (two-sided)

^b^median value

^c^75th percentile

^d^Luminal A - HER2^+^, PgR^+^, HER2^-^, CK5/6^-^; Luminal B - ER^+^, PgR^+^, HER2^+^, CK5/6^-^; HER2^+^ - ER^-^, PgR^-^, HER2^+^ CK5/6^-^; Basal-like - ER^-^, PgR^-^, HER2^-^, CK5/6^+^

The study has been approved by the Ethics Committee at the Centre of Oncology, Krakow, Poland (date of issue 14.02.2006).

### Preparation of Tissue

MVD and p53 status were assessed on formalin fixed and paraffin-embedded sections. Before staining sections were deparaffinized in xylenes and rehydrated trough graded alcohol steps. To quench the endogenous peroxidase activity, the slides were treated with 0.3 % hydrogen peroxide in alcohol at 95 % for 30 min. and then washed in distilled water for 10 min. MVD and p53 status were evaluated IHC.

### Immunohistochemistry

In both IHC staining, for antigen unmasking, 50 min incubation in Target Retrieval Solution (TRS), (pH = 6.1, DAKOCytomation, Glostrup, Denmark), preheated to 96°C was applied. The following primary monoclonal antibodies were used: antiCD34 (class II, clone QBEnd 10, DakoCytomation, Glostrup, Denemark, dilution 1:50) and NCL-p53-1801 (Novocastra, UK, dilution 1:40). In both cases, whole night incubation with diluted primary antibody at 4°C in humidity chamber was carried out. The antigen-primary antibody complex was detected with EnVision + ® + System-HRP (DAB) system (DAKOCytomation, Glostrup, Denmark). Peroxidase was visualised using 0.01 % 3.3-diaminobenzidine tetrahydrochloride (DAB) and 0.015 % hydrogen peroxide. The slides were counterstained with Mayer’s hematoxylin. For negative control, tris buffered saline (TBS) was substituted for each primary antibody. Positive control in both stainings includes breast carcinomas exhibiting high expression of each marker.

### Evaluation of IHC Staining

**Fig. 1:**
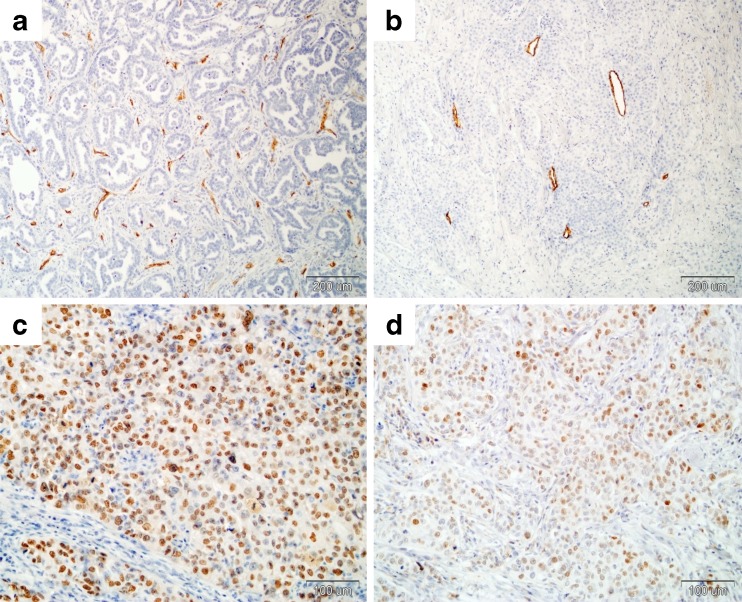
Immunohistochemical staining representative examples of CD34-positive endothelial cells of blood vessels and p53 positive nucleus in breast tumour tissues. Cancers with higher **a** and lower **b** vascularization (magnification 100X) or stronger **c** and weaker **d** p53 protein expression (magnification 200X)

Each section was assessed blind without any knowledge of the patient’s previous investigations or treatment outcome. MVD density was determined as previously described by Weidner et al. [4] using Olympus microscope (Olympus Optic Co., Ltd, Tokyo, Japan). First, the five areas of invasive component of tumor with the highest number of microvessels (tumor “hot spot”) were identified at low magnification (X 40). Then, in each area recognized, the number of individually stained vessels was counted at X 200 magnification (0.29 mm^2^ per field) and MVD was calculated per 1 mm^2^ (Fig. 1a and b). A single microvessel was defined as any brown immunostained endothelial cell that was separated from adjacent microvessel tumor cell and other connective tissue elements. MVD was expressed as the mean number of microvessels per mm^2^, taken the average from the five “hot-spots” counts.

p53 immunoreactivity was restricted to the nucleus (Fig. 1c and d). For each section at least 1000 tumors cells were counted at X 400 magnification. p53 labelling index (p53LI) was calculated as the percentage of p53 nuclear immunopositive cells. The sections were graded as positive if p53LI was >10.0 % of stained tumor cells according to the value used by other authors [21, 23, 27].

### Statistical Analysis

Descriptive statistics was used to determine mean and median values of continuous variables (MVD, p53LI) and standard errors of means (SE). Mann-Witney *U* test was used to establish the significance of differences between means of continuous variables. Associations between categorical variables were analyzed using Pearson *χ* ^2^ test. The primary endpoint for the study was DFS, defined as the time from surgery to the first observation of tumor progression (locoregional recurrence, distant recurrence or second malignancy). The median duration of DFS was calculated using the Kaplan-Meier method [28]. Comparisons between groups were made using log-rank test. Multivariate analysis was carried out using the Cox proportional hazards model. Two-sided *P* values of <0.05 were considered significant. All statistical analyses were carried out using Statistica v.9.0 program.

## Results

### Patients

Patient characteristic is shown in Table 1 and has also previously presented in details [3]. Briefly, there were 172 women in age from 32 to 78 years (with mean and median values 52.8 years ± 0.67 and 53 years, respectively) with breast cancer in clinical stage T1N1 (54.7 %), T1N2 (23.3 %), T2N1 (18.0 %) and T2N2 (4.0 %). All women received anthracyclines as adjuvant chemotherapy according to two regimes: doxorubicin/cyclophosphamide (AC) or cyclophosphamide/5-fluorouracil/doxorubicin (CAF). Of the initial 172 patients, complete follow-up was available in 167. The median follow-up time was 36 months (range 3–64 months). From 167 women, 165 (98.8 %) were alive at the time of the study, 2 (1.2 %) died from breast cancer after 19 and 14 months from surgery. Tumor progression was observed in 20 patients (12.1 %).

### MVD, p53 Status and Clinical and Histopathological Data

The mean and median values of MVD were 182.9 vessel/mm^2^ ± 4.5 (SE) and 173.4 vessel/mm^2^ (range: 80.8 – 313.3 vessel/mm^2^), respectively (Fig. 1a and b). Tumors were classified into lower and higher MVD groups using the 75th percentile (214.8 vessel/mm^2^) as cutoff point. There were 123 tumors (71.5 %) with lower MVD and 49 cancers (28.5 %) with higher MVD. The proportion of tumors with higher MVD significantly increased in N2 tumors (*P* = 0.000) and in ER (*P* = 0.046) or PgR (*P* = 0.029) negative tumors (Table 1). The same was true when MVD was analyzed as continues variable (data not shown). We did not observe any other association between MVD and other clinicopathological variables (Table 1).

The mean and median values of p53LI were 11.5 % ± 1.9 (SE) and 0.0 % (range: 0.0–90.7 %), respectively (Fig. 1c and d). p53 status was positive (p53LI > 10.0 %) in 50 tumors (29.1 %). p53 positivity was significantly associated with higher histological grade (*P* = 0.000), steroid receptors negativity (*P* = 0.000), CK5/6 positivity (*P* = 0.026), TOPOIIα overexpression (*P* = 0.005) and higher proliferation rate (*P* = 0.001) (Table 1). The proportion of p53 positive tumors was also significantly higher (*P* = 0.001) in HER2^+^ and basal-like breast cancer immunophenotypes than in luminal A and B subtypes (Table 1). The same relations were observed when p53LI was analyzed as continues variable (data not shown). No other significant interactions between p53 status and further clinical and histopathological parameters were observed (Table 1).

### Univariate Influence of Markers on DFS

**Fig. 2:**
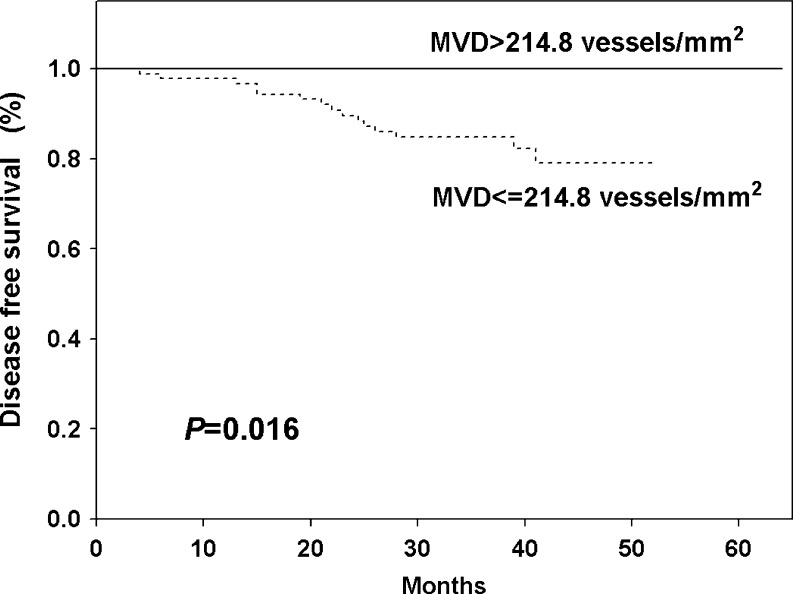
Kaplan-Meier curve for disease free survival depending on microvessel density level

**Fig. 3:**
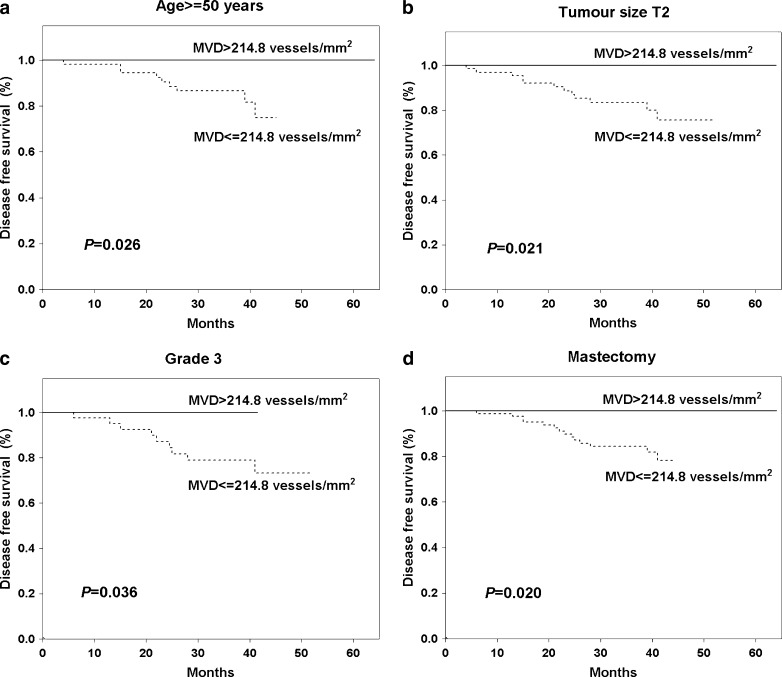
Kaplan-Meier curves for disease free survival in relation of microvessel density level in respect to patient’s age **a**, tumor size **b**, histological grade **c** and type of surgery **d**

**Fig. 4:**
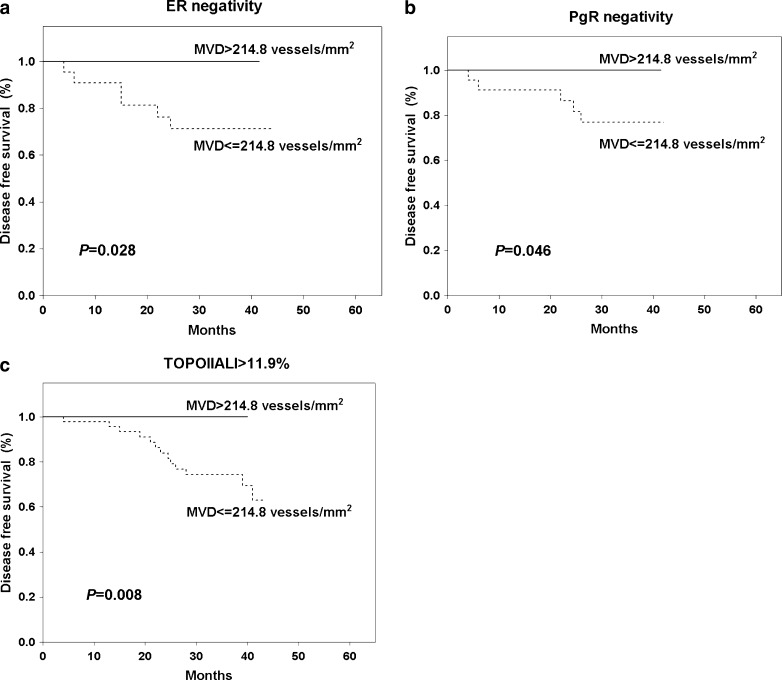
Kaplan-Meier curves for disease free survival in relation of microvessel density level in respect to estrogen receptor **a**, progesterone receptor **b** and topoisomerase IIα expression **c**

To examine the relation between the MVD and DFS the patients were divided into two groups: those with lower and those with higher MVD tumors, initially on the basis of mean (182.9 vessel/mm^2^) and median (173.4 vessel/mm^2^) values. Using these cutoff points, there were no significant differences in DFS observed between patients with lower and higher MVD tumors (Table 2). Additionally, we tested 67th, 75th, 80th and 90th percentiles as cutoff points. The value of 214.8 microvessels/mm^2^ i.e. 75th percentile had the most potent statistical value as a cutoff point (Table 2). All women (n = 49) with higher MVD (>214.8 microvessels/mm^2^) survived 5 years without any evidence of cancer disease, whereas patients with lower MVD were characterised by significantly (*P* = 0.016) worse DFS (79.7 %) (Fig. 2). When Kaplan-Meier curves were analyzed with consideration to patient subgroups, significant differences in DFS were obtained for older women (*P* = 0.026, Fig. 3a), patients with T2 (*P* = 0.021, Fig. 3b) and grade 3 tumors (*P* = 0.036, Fig. 3c) and for women undergoing mastectomy (*P* = 0.020, Fig. 3d). This effect was also observed only in the group of patients with ER (*P* = 0.028, Fig. 4a) or PgR negative (*P* = 0.046, Fig. 4b) and TOPOIIα overexpressed tumors (*P* = 0.008, Fig. 4c).

**Table 2:** Univariate Cox proportional hazard model for MVD different cutoff points and disease free survival of 167 breast cancer patients treated with adjuvant anthracycline based chemotherapy

MVD cutoff point	Response	HR	95 % CI	*P*-value
	N (%)			
Median value:				
Higher MVD > 173.4 vessel/mm^2^	77/85 (90.6)	1.000		
Lower MVD ≤ 173.4 vessel/mm^2^	65/82 (79.3)	1.047	0.344–2.653	0.402
Mean value:				
Higher MVD > 182.9 vessel/mm^2^	64/71 (90.1)	1.000		
Lower MVD ≤ 182.9 vessel/mm^2^	77/96 (80.2)	1.655	0.210–1.742	0.362
67th percentile:				
Higher MVD > 202.0 vessel/mm^2^	49/52 (94.2)	1.000		
Lower MVD ≤ 202.0 vessel/mm^2^	91/115 (79.1)	3.307	0.075–1.212	0.081
75th percentile:				
Higher MVD > 214.8 vessel/mm^2^	49/49 (100.0)	1.000		
Lower MVD ≤ 214.8 vessel/mm^2^	98/123 (79.7)	17.184	0.004–0.897	0.016
80th percentile:				
Higher MVD > 226.6 vessel/mm^2^	35/35 (100.0)	1.000		
Lower MVD ≤ 226.6 vessel/mm^2^	107/132 (81.1)	19.144	0.002–1.515	0.035
90th percentile:				
Higher MVD > 265.0 vessel/mm^2^	23/23 (100.0)	1.000		
Lower MVD ≤ 265.0 vessel/mm^2^	120/144 (83.3)	24.680	0.000–15.211	0.187

*HR* hazard ratio, *CI* confidence interval, *MVD* microvessel density

**Fig. 5:**
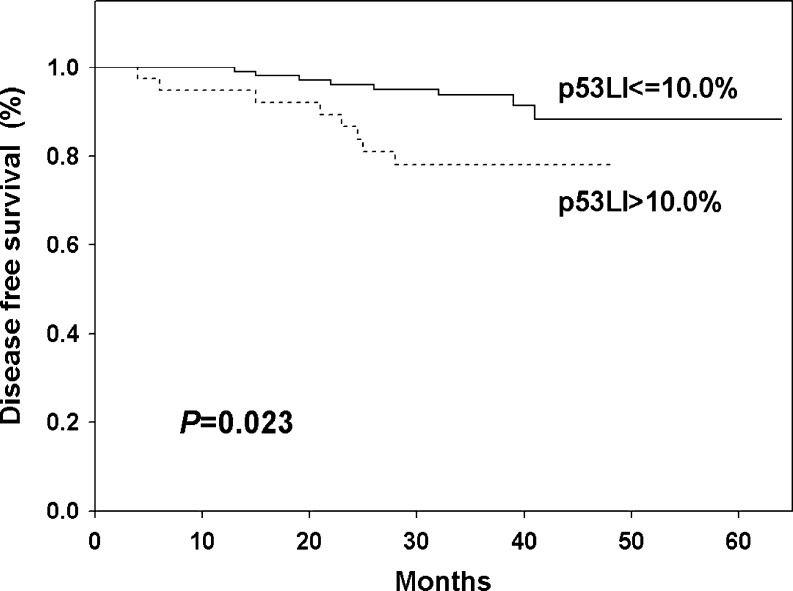
Kaplan-Meier curve for disease free survival depending on p53 status

**Fig. 6:**
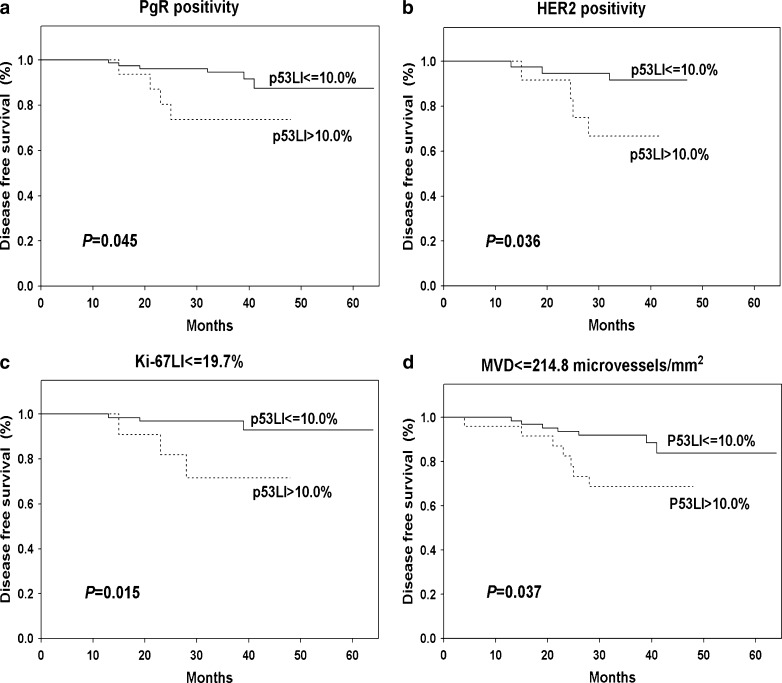
Kaplan-Meier curves for disease free survival in relation of p53 status in respect to progesterone receptor status **a**, HER2 status **b**, proliferation rate **c** and microvessel density **d**

The significant relation between p53 status and DFS was observed. Patients with p53 negative tumors ware characterized by significantly (*P* = 0.023) higher DFS (88.3 %) in comparison to those with p53 positive cancers (78.7 %) (Fig. 5). The impact on DFS was more evident in the group of patients with PgR (*P* = 0.045, Fig. 6a) and HER2 positive tumors (*P* = 0.036, Fig. 6b) with lower proliferation rate (*P* = 0.015, Fig. 6c) and lower MVD (*P* = 0.037, Fig. 6d).

### Multivariate Analysis for DFS

Previously, in this patient cohort, in the univariate analysis we have found significantly higher DFS for patients with tumors presenting lower histological grade and lower TOPOIIα [3]. Significant differences in DFS were also observed between cancer immunophenotypes and tumor subgroups distinguished according to TOPOIIα expression and HER2 status as well as cancer subtypes according to TOPOIIα expression and grade [3]. Therefore to evaluate prognostic potential of MVD and p53 status we performed multivariate Cox regression analysis according to two models. In model I, including grade, TOPOIIALI, MVD and p53 status, patients were dichotomised according to single variables (Table 3). In this model TOPOIIALI and MVD were identified as independent prognostic parameters. In model II, MVD and p53 status were analyzed together with: (1) cancer immunophenotype, (2) tumor subgroups distinguished according to TOPOIIα expression and HER2 status and (3) cancer subtypes according to TOPOIIα expression and grade (Table 3). In this model MVD and cancer subtype according to TOPOIIα expression and grade were independent prognostic variables.

**Table 3:** Multivariate Cox regression analysis

	HR	95 % CI	*P*- value
Model I			
Grade:			
1 + 2	1.000		
3	1.271	0.438–3.494	0.648
TOPOIIALI^a^:			
≤11.9 %	1.000		
>11.9 %	6.284	0.188–21.011	0.021
MVD^b^:			
>214.8 vesels/mm^2^	1.000		
≤214.8 vesels/mm^2^	13.695	0.007–0.807	0.034
p53LI:			
≤10.0 %	1.000		
>10.0 %	2.035	0.804–5.151	0.136
Model II			
MVD^b^:			
>214.8 vesels/mm^2^	1.000		
≤214.8 vesels/mm^2^	18.357	0.005–0.614	0.040
p53LI:			
≤10.0 %	1.000		
>10.0 %	1.941	0.767–4.910	0.163
Breast cancer immunofenotype:			
Luminal A^c^	1.000		
Other^c^	1.657	0.592–4.639	0.338
Breast cancer subtypes according to TOPOII α expression and HER2 status:			
TOPOIIALI *≤* 11.9 % and HER2^-^	1.000		
Other^d^	3.849	0.571–25.896	0.169
Breast cancer subtypes according to TOPOII α expression and grade:			
TOPOIIALI *≤* 11.9 % and G1 + G2	1.000		
Other^e^	18.357	1.561–75.897	0.021

*HR* hazard ratio, *CI* confidence interval, *TOPOIIALI* topoisomerase IIα labelling index, *MVD* microvessel density, *p53LI* p53 labelling index

^a^median value

^b^75th percentile

^c^Luminal A-ER^+^, PgR^+^, HER2^-^, CK5/6^-^; Luminal B-ER^+^, PgR^+^, HER2^+^, CK5/6^-^; HER2^+^-ER^-^, PgR^-^, HER2^+^, CK5/6^-^; Basal-like-ER^-^, PgR^-^, HER2^-^, CK5/6^+^

^d^TOPOIIALI *≤* 11.9 % and HER2^+^, TOPOIIALI > 11.9 % and HER2^+^, TOPOIIALI > 1.9 % and HER2^-^

^e^TOPOIIALI *≤* 11.9 % and G3, TOPOIIALI > 11.9 % and G3, TOPOIIALI > 11.9 % and G1 + G2

## Discussion

In the present retrospective study, immunohistochemical staining was used to determine the prognostic significance of MVD and p53 status in a homogenous patient cohort (T1-T2, N1-N2, M0) who received anthracyclines as adjuvant chemotherapy. In this study we have shown, according to our best knowledge for the first time, that all patients with higher MVD tumors survived 5 years without any evidence of cancer disease, whereas DFS for patients with lower MVD cancers was 79.7 % (median follow-up 36 months). The role of MVD as independent prognostic parameter was also confirmed in multivariate Cox regression analysis. A significant difference in DFS was also observed between women with p53 negative tumors (88.3 %) and patients with p53 positive cancers (78.7 %), however, in multivariate Cox regression analysis p53 status did not reach statistical significance.

In this study, higher MVD level was a favourable prognostic factor for patients DFS after anthracyclines based adjuvant chemotherapy. This finding is in concordance with results obtained by several other authors. Ioachim et al. [16] have analyzed MVD, using Factor VIII as endothelial marker, in the group of 82 patients with T1-T3, N0-N+, M0 breast cancer. They have found, similarly to us, significant correlation between higher MVD and increased relapse free survival (RFS) and overall survival (OS). In turn, Protopapa et al. [17] in a small group of 26 premonopausal women with ductal invasive, 2–5 cm in diameter carcinomas, treated before or after mastectomy with anthracyclines, have shown longer survival in the group of patients with higher MVD (assessed by Masson’s Trichrome technique) tumors. Gunel et al. [18] in 42 early breast cancer patients (T1-2, N0-2, M0), revealed that anthracycline based adjuvant chemotherapy was particularly effective in lymph node positive breast cancer patients with increased angiogenesis. One of possible explanations of positive correlation between higher MVD and better results of anthracyclines treatment is related with the mechanisms of these drugs’ action. A principal mechanism of anthracycline cytotoxic effects is their ability to intercalate into DNA and to inhibit topoisomerase II activity. Along with topoisomerase inhibition, anthracyclines stimulate the formation of reactive oxygen species (ROS), which at high level, can significantly contribute to the cytotoxic activity of these drugs through induction of cell death, apoptosis and senescence [29]. Therefore, in well vascularized tumors, with good oxygen access, the production of ROS may be even 2-fold greater [30] and thus cytotoxicity may be also greater. It has been also suggested that ROS, as signalling molecules, stimulate angiogenesis process via induction of redox sensitive gene expression (VEGF, matrix metalloproteinases, urokinase plasminogen activator) [31]. Taking into account all these facts, it is a kind of positive feedback loop, in which high level of ROS (result of anthracycline acction) stimulates higher cytotoxicity and activates new vessel formation that is responsible for even higher ROS level. Higher antharcycline sensitivity in more vascularised tumors may be also related with increased cytotoxic agents access to tumor cells.

By contrast, there are three studies, in which reverse correlation between MVD and the patients survival after anthracycline based adjuvant chemotherapy was found. In the group of 215 women with T1-T3, N+, M0 breast cancer treated with four cycles of doxorubicin–containing therapy followed by high doses of cyclophosphamide/cisplatin/carmustine, Nieto et al. [8] have found significantly longer RFS and OS for patients with lower MVD tumors. Similar relation was found in the studies of Tas et al. [9] in the group of 120 breast cancer (T1-T3, N0-3, M0) patients treated with CAF or cyclophosphamide/methotrexate/fluorouracil (CMF) and of Viens et al. [10] who studied 135 women with breast cancer (T0-3, N1-3, M0) after CAF. The authors explain these results by important role of angiogenesis in tumor growth, invasion and metastasis [7, 32].

Moreover, there are several papers [11–15], in which no significant relation between MVD level and treatment outcome after anthracyclines based adjuvant chemotherapy was obtained. The discordances in results concerning the MVD prognostic value may be a consequence of many reasons. First of all, in many studies, great heterogeneity in adjuvant chemotherapy schedule and tumor clinical stage was observed [9, 12, 13]. Besides, some studies, in which higher MVD level was related with worse treatment outcome, analyzed relatively long term follow-up (9 years in the study of Nieto et al. [8], and 5 years in Viens et al. [10]). As in our study the median follow-up was 36 months, it could be speculated that the prognostic significance of MVD may change during follow-up time and higher MVD level is related with good prognosis within 3 years after initial treatment. Another reason of contrary results respecting MVD prognostic potential is connected with differences in the methodology and criteria of MVD evaluation. These differences concern the use of different antibodies (Factor VIII [12, 15, 16], CD-31 [8, 10, 11, 14], CD-34 [9, 13], endoglin [11]) characterised by different specificity [33], the method applied to count vessels (hot spot method [8, 11, 12, 14–16] or Chalkley count [13]) and “hot spot” technique modification concerning the number of analyzed hot spots, which can vary from 1 (first hot-spot method) [9], trough 3 [12–14, 16] to 5 [10] or more [15]. Moreover, there are some discrepancies between authors concerning cutoff points used to distingiush lower and higher MVD tumor subgroups. We identified cutoff point at the level of 75th percentile which is quite often found as statistically most potent by other authors [8, 33]. In other reports median [9–12, 14, 15], or tertiles [13, 18] were used as a cutoff point.

In the present study, MVD did not correlate with TOPOIIα expression, nevertheless on the basis of these two biomarkers we were able to identify a subgroup of patients without any evidence of early recurrence after anthracycline treatment [3]. As we have shown, higher MVD (in our study, “low risk” patient group) was significantly related with lower N stage, hormonal receptors positivity and cytokeratin 5/6 negativity. Previously we have shown that lower TOPOIIα expression in tumors (indicated also patients with good prognosis) was significantly related with lower histologial grade, hormonal receptor positivity, cytokeratin 5/6 negativity, p53 negativity, lower proliferation rate and luminal A immunophenotype [3]. All these observations may suggest that these two biomarkers: MVD and TOPOIIα expression, allow for identification of two different subgroups of patients, both characterised by good prognosis and hence confirm the hypothesis that different molecular mechanisms are responsible for sensitivity or resistance to chemotherapy [29].

In our study p53 negativity was significantly related to longer DFS. This finding is in accordance with the results of other authors [21–24]. They have reported statistically significant association between p53 immunopositivity and lack of response to adjuvant anthracycline based chemotherapy. Also in experimental studies performed on cell lines [19] or on mice with p53 lacking tumors [20] this effect was seen. These results can be explained in part by the fact that certain p53 gene can up-regulate the expression of multidrug resistance gene (*MDR1*) via stimulation of gene promoter [34]. Moreover, p53 has the key role in apoptosis activation. However, some reports [25–27] failed to show a p53 prognostic potential determined by immonohistochemistry for response to adjuvant chemotherapy with anthracyclines. Lack of association between p53 mutations and p53 immunoreactivity may explain, in part, these contradictionary results. Lack of immunostaining is particularly frequent in tumors with p53 nonsense mutations [35]. Mutations in p53 may have also different biological effects. Some studies have shown that mutations in L2 and L3 domains, which are critical regions responsible for DNA binding, were associated with poor prognosis in breast cancer and correlated with resistance to doxorubicin [26, 35]. In turn, Bug et al. [36] have shown that even closely related anthracyclines induce the synthesis of different, opposing transcripts from p53 locus. Therefore, the minor differences in treatment schedule can influence the results concerning p53 prognostic role.

## Conclusion

In the present study we have shown, according to our best knowledge for the first time, that all patients with higher MVD tumors are characterized by a very good prognosis during the first 3 years after completion of adjuvant anthracycline based treatment. Additionaly, these results suggest that MVD may have also predictive potential, because women with low MVD tumors are possible candidates for alternative than anthracyclines adjuvant chemotherapy. However, we plan to verify presented results in additional analysis with longer follow-up.
